# Model-based cost-effectiveness analysis of B-type natriuretic peptide-guided care in patients with heart failure

**DOI:** 10.1136/bmjopen-2016-014010

**Published:** 2016-12-26

**Authors:** Syed Mohiuddin, Barnaby Reeves, Maria Pufulete, Rachel Maishman, Mark Dayer, John Macleod, Theresa McDonagh, Sarah Purdy, Chris Rogers, William Hollingworth

**Affiliations:** 1 School of Social and Community Medicine, University of Bristol, Bristol, UK; 2 School of Clinical Sciences, University of Bristol, Bristol, UK; 3 NHS Practice, Taunton and Somerset NHS Trust, Somerset, UK; 4 Faculty of Life Sciences and Medicine, King's College London, London, UK

**Keywords:** B-type natriuretic peptide, Cost-effectiveness, Markov model, Survival analysis

## Abstract

**Objective:**

Monitoring B-type natriuretic peptide (BNP) to guide pharmacotherapy might improve survival in patients with heart failure with reduced ejection fraction (HFrEF) or preserved ejection fraction (HFpEF). However, the cost-effectiveness of BNP-guided care is uncertain and guidelines do not uniformly recommend it. We assessed the cost-effectiveness of BNP-guided care in patient subgroups defined by age and ejection fraction.

**Methods:**

We used a Markov model with a 3-month cycle length to estimate the lifetime health service costs, quality-adjusted life years (QALYs) and incremental net monetary benefits (iNMBs) of BNP-guided versus clinically guided care in 3 patient subgroups: (1) HFrEF patients <75 years; (2) HFpEF patients <75 years; and (3) HFrEF patients ≥75 years. There is no evidence of benefit in patients with HFpEF aged ≥75 years. We used individual patient data meta-analyses and linked primary care, hospital and mortality data to inform the key model parameters. We performed probabilistic analysis to assess the uncertainty in model results.

**Results:**

In younger patients (<75 years) with HFrEF, the mean QALYs (5.57 vs 5.02) and costs (£63 527 vs £58 139) were higher with BNP-guided care. At the willingness-to-pay threshold of £20 000 per QALY, the positive iNMB (£5424 (95% CI £987 to £9469)) indicates that BNP-guided care is cost-effective in this subgroup. The evidence of cost-effectiveness of BNP-guided care is less strong for younger patients with HFpEF (£3155 (−£10 307 to £11 613)) and older patients (≥75 years) with HFrEF (£2267 (−£1524 to £6074)). BNP-guided care remained cost-effective in the sensitivity analyses, albeit the results were sensitive to assumptions on its sustained effect.

**Conclusions:**

We found strong evidence that BNP-guided care is a cost-effective alternative to clinically guided care in younger patients with HFrEF. It is potentially cost-effective in younger patients with HFpEF and older patients with HFrEF, but more evidence is required, particularly with respect to the frequency, duration and BNP target for monitoring. Cost-effectiveness results from trials in specialist settings cannot be generalised to primary care.

**Strengths and limitations of this study**

- We combined recent individual patient data meta-analyses of 2000 heart failure (HF) patients participating in several randomised controlled trials (RCTs) and evidence on the costs of care from routine data in a cost-effectiveness model.
- We investigated the cost-effectiveness of B-type natriuretic peptide (BNP)-guided care in subgroups of HF patients which were not reported in the original RCT publications.
- We conducted sensitivity analyses to estimate the uncertainty around our results and identify patient subgroups where further evidence is needed.
- We used a simplified model structure as the majority of RCTs do not measure or report changes in functional status at follow-up.
- None of the RCTs provided evidence on utility scores; hence, we used scores reported elsewhere in the HF literature which may not be representative of patients eligible for BNP monitoring.

## Introduction

Heart failure (HF) is a major and growing public health problem worldwide. HF is associated with high risks of hospitalisation and mortality, making it one of the most costly conditions to manage. Global estimates indicate that HF results in US$65 billion direct care costs and US$43 billion in lost productivity annually.^1^ In the UK, ∼500 000 people live with HF,^2^ and this figure is likely to rise as the population continues to age. Each year, HF is the primary diagnosis in over 150 000 hospital episodes in the UK; many of these are emergency admissions.^2^ Healthcare costs increase sharply at the end of life and are dominated by hospital care.^3^ The case for many novel interventions in HF is based on the expectation that the upfront costs will be justified in the longer term by improved patient outcomes and/or savings due to reduced hospitalisations.^4^ ^5^

Pharmacological treatment for HF includes ACE inhibitors, angiotensin receptor blockers, β blockers and mineralocorticoid receptor antagonists. Achieving optimal pharmacotherapy for HF is challenging and complicated by potential side effects such as renal failure and hypotension. Emerging evidence suggests that monitoring serum B-type natriuretic peptide (BNP) biomarkers can guide pharmacotherapy and improve survival.^6^ ^7^ BNP is a neurohormone secreted primarily from the left ventricle of the heart in response to changes in pressure that occur when HF develops and worsens. In 2010, clinical guidelines^8^ in England and Wales recommended specialist monitoring of BNP in patients recently admitted to hospital, but also called for further research on cost-effectiveness. More recent North American guidelines^9^ considered the value of serial BNP measurement to be not well established. Evidence on the cost-effectiveness of BNP-guided care in HF includes economic evaluations conducted alongside randomised controlled trials (RCTs)^10^ ^11^ and model-based analyses^12–14^ synthesising costs and outcomes over the lifetime of patients.

We conducted a model-based cost-effectiveness analysis of BNP-guided care in patients with HF. Our analysis differs from previous economic evaluations in two ways. First, we exploited recent individual patient data (IPD) meta-analyses^6^ ^7^ in estimating the relative effect of BNP-guided care. Among the advantages of IPD meta-analysis is the opportunity to investigate the (cost-)effectiveness of BNP-guided care in subgroups of patients which are not analysed consistently or not reported in the original RCT publications. Second, we used linked data from the Clinical Practice Research Database (CPRD; http://www.cprd.com), Hospital Episode Statistics (HES; http://www.hscic.gov.uk/hesdata) and Office for National Statistics (ONS; http://www.ons.gov.uk) to inform key parameters of the model. In particular, we used these data to estimate the National Health Service (NHS) costs of care for patients with HF who are stable and treated in primary care compared with those who are admitted to hospital. Our objective was to synthesise the evidence on the cost-effectiveness of BNP-guided care in subgroups of the recently hospitalised HF population defined by age and left ventricular ejection fraction (LVEF) status.

## Methods

### Overview of the model

**Figure 1:**
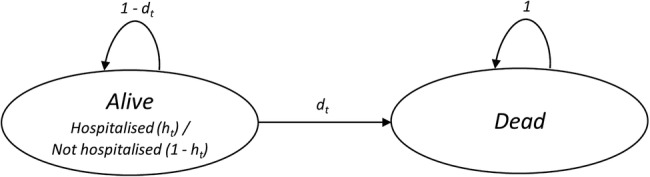
Markov model of disease progression.

We compared specialist-led BNP-guided care with specialist-led clinically guided care in recently hospitalised patients with HF with a reduced ejection fraction (HFrEF) or preserved ejection fraction (HFpEF). Two RCTs^15^ ^16^ included a third arm where usual care was provided by a primary care physician, but we focused on specialist-led care in common with the IPD meta-analyses of RCTs.^6^ ^7^ We used a simple Markov process consisting of two health states: *Alive* and *Dead* (figure 1), which is similar to the structure of a previous cost-effectiveness model that was used to develop NICE clinical guidelines on the management of HF^8^ and later updated with additional RCT evidence.^14^

We tracked the probabilities of death (d_t_) and hospitalisation (h_t_), which varied with time in the model. We did not model the interaction between the number of hospitalisations and the subsequent risk of death. This was a pragmatic decision as RCTs do not report mortality conditional on the number of hospitalisations. We chose a cycle length of 3 months to track changes in outcomes and resource use, as BNP was monitored every 3 months during follow-up in a number of RCTs^16–18^ and mortality differences have emerged by 3 months in IPD meta-analyses.^6^ ^7^ We assumed that transitions between health states occur halfway through each cycle.

We estimated the average NHS costs and quality-adjusted life years (QALYs) of a hypothetical cohort of 1000 patients over their lifetime. Recent IPD meta-analyses of RCTs^6^ ^7^ have explored the relative effectiveness of BNP-guided care in subgroups of the recently hospitalised HF population defined by age (<75/≥75 years) and LVEF (≤45%/>45%) status. The evidence that BNP-guided care is effective is strongest in younger patients (<75 years) and in those with HFrEF. However, there is no evidence to suggest that BNP-guided care is beneficial in older patients (≥75 years) with HFpEF (HR 1.56 (95% CI 0.90 to 2.70)).^6^ Therefore, we estimated the cost-effectiveness of BNP-guided care in three patient subgroups: (1) HFrEF patients <75 years; (2) HFpEF patients <75 years; and (3) HFrEF patients ≥75 years. Based on data from the IPD meta-analysis,^6^ we used the mean ages of 65 and 81 years at the inception of treatment for the age-subgroups <75 and ≥75 years, respectively. We tracked outcomes for a period of 30 years for the age-subgroup <75 and 15 years for the age-subgroup ≥75 years. This equated for both strategies that more than 99% of patients analysed have died.

We calculated QALYs by multiplying the health state utility score by the time spent in that state.^19^ Costs and QALYs were discounted at an annual rate of 3.5%.^20^ We estimated the cost-effectiveness of BNP-guided care based on the incremental net monetary benefit (iNMB):
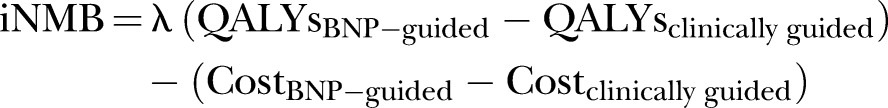


where λ represents the maximum amount that the NHS is ‘willing to pay’ to gain one QALY. We used the lower NICE threshold (λ=£20 000) in calculating iNMB.^21^ An iNMB value >0 would indicate that BNP-guided care is cost-effective. We present cost-effectiveness acceptability curves (CEACs) to demonstrate how the NHS willingness-to-pay threshold affects the probability that BNP-guided care is considered cost-effective.^22^ We also present incremental cost-effectiveness ratios (ICERs).

### Model parameters

We used four sets of parameters (tables 1 and 2): (1) baseline probabilities of hospitalisation or death in the clinically guided group; (2) relative treatment effects (hazard ratios (HRs) and relative risks (RRs)), which determine how the baseline probabilities differ in the BNP-guided group; (3) utilities, which represent the health-related quality of life of patients in each state; and (4) NHS costs incurred in each state. The sources of these data are described below.

**Table 1:** Transition probability parameters used in the model

Parameter	Mean estimate	Distribution	Source
Baseline monthly hazard rate of all-cause mortality for the first 8 years of the model (<75 years)	0.009	LN (−4.718, 0.012 SE)	CPRD-ONS
HR (≥75 vs <75 years) of all-cause mortality for the first 8 years of the model	2.801	LN (1.030, 0.014 SE)	CPRD-ONS
3-monthly risk of all-cause mortality in general population	Age variant	Fixed	ONS^23^
RR (HF patients vs general population) of all-cause mortality	3.14	β (199, 94)* HF/ β (176, 410)* gen. pop.	van Jaarsveld *et al*^24^
RR (HFpEF patients vs HFrEF patients) of all-cause mortality	0.78	β (766, 2865)* HFpEF/ β (584, 1621)* HFrEF	Nichols *et al*^25^
BNP HR of all-cause mortality for HFrEF patients <75 years	0.68	LN (−0.386, 0.177 SE)	Brunner-La Rocca *et al*^6^
BNP HR of all-cause mortality for HFpEF patients <75 years	0.76	LN (−0.274, 0.487 SE)	Brunner-La Rocca *et al*^6^
BNP HR of all-cause mortality for HFrEF patients ≥75 years	0.87	LN (−0.139, 0.148 SE)	Brunner-La Rocca *et al*^6^
Baseline monthly hazard rate of all-cause hospitalisation (<75 years)	0.066	LN (−2.711, 0.008 SE)	CPRD-ONS
HR (≥75 vs <75 years) of all-cause hospitalisation	1.248	LN (0.222, 0.010 SE)	CPRD-ONS
BNP HR of all-cause hospitalisation	0.94	LN (−0.062, 0.062 SE)	Troughton *et al*^7^

*β parameters were determined empirically.

CPRD, Clinical Practice Research Database; HES, Hospital Episode Statistics; LN, log normal; ONS, Office for National Statistics; RR, relative risk.

**Table 2:** Utility, resource use and cost parameters used in the model

Parameter	Mean estimate	Distribution	Source
HF utility score when hospitalised	0.66 (0.26 SD)	β (7321, 3772)*	Reed *et al*^26^
HF utility score when not-hospitalised	0.77 (0.23 SD)	β (7978, 2383)*	Reed *et al*^26^
Duration of hospitalisation (days)	13.21 (0.39 SE)	γ (1148.29, 0.01)*	CPRD-HES^3^
3-monthly cost when hospitalised (age <75 years)	£9104 (349.61 SE)	γ (678.06, 13.43)*	CPRD-HES^3^
3-monthly cost when not-hospitalised (age <75 years)	£682 (23.72 SE)	γ (827.17, 0.82)*	CPRD-HES^3^
3-monthly cost when hospitalised (age ≥75 years)	£8057 (192.77 SE)	γ (1746.96, 4.61)*	CPRD-HES^3^
3-monthly cost when not-hospitalised (age ≥75 years)	£569 (14.52 SE)	γ (1536.51, 0.37)*	CPRD-HES^3^
Clinically guided unscheduled outpatient visits (24 months)	1.10 (0.13 SE)	γ (71.60, 0.02)*	PRIMA^27^
BNP-guided unscheduled outpatient visits (24 months)	1.40 (0.14 SE)	γ (94.52, 0.02)*	PRIMA^27^
BNP-guided additional cost of medications (18 months)	£58.32 (6.20 SE)	γ (88.42, 0.66)*	TIME-CHF^11^
Unit cost of an outpatient visit	£123	Fixed	DoH^28^
Unit cost of a BNP test	£25	Fixed	NICE^29^

*β and γ parameters were determined using the methods of moments described elsewhere.^30^

CPRD, Clinical Practice Research Database; HES, Hospital Episode Statistics.

### Baseline probabilities: mortality

We used routinely collected CPRD-ONS linked data from April 2005 up to the censoring date of April 2014, including 52 122 patients with a HF hospital admission (identified by ICD-10 diagnosis code), to estimate the monthly mortality rate in clinical practice in the absence of BNP-guided care. The mean (SD) age of the patients at admission was 77.9 (12) years, and 49% were female. This allowed us to estimate survival over a longer time period than is available from RCTs. We excluded patients who died in hospital or within 7 days of discharge, as they would be unlikely to be eligible for BNP-guided care, and a small minority who had frequent BNP testing indicating that they were already receiving BNP-guided care. Time between discharge from first HF admission to all-cause mortality and all-cause rehospitalisation were calculated. Parametric survival models (assuming an Exponential distribution and a Weibull distribution) were fitted to the data fitting age (dichotomised into <75 and ≥75 years) to obtain estimates of age-specific all-cause hazard rates. These survival analyses were carried out in Stata V.14.0. In our primary analyses, we used the exponential hazard rate of 0.009 to estimate survival for younger patients for the first 8 years of the model. In a sensitivity analysis (SA1), we used the Weibull hazard rate of 0.017 together with the ancillary parameter estimate of 0.842 to estimate survival for younger patients for the first 8 years of the model.

Beyond the initial period, we used age-specific and sex-specific ONS UK life-tables^23^ to estimate survival, assuming that two-thirds of patients were male^7^ and inflating population mortality for the HF population using a RR derived from an observational study.^24^ van Jaarsveld *et al*^24^ report 32% survival at 7-year for 293 incident HF cases diagnosed in the Netherlands between 1993 and 1998, compared with 70% survival among 586 age and sex-matched controls without HF. These 7-year survival probabilities were converted to 3-monthly survival probabilities. The majority of patients recruited to trials had HFrEF. After adjusting for age, gender and other covariates, mortality has been demonstrated to be lower in patients with HFpEF.^25^ ^31^ Therefore, we adjusted survival in the HFpEF subgroup evaluated in our model, using results from a cohort of more than 6500 patients hospitalised for HF which reported an adjusted 1-year mortality RR of 1.25 (95% CI 1.12 to 1.41) in patients with HFrEF versus HFpEF.^25^

### Relative effects: mortality

We used an IPD meta-analysis to estimate the relative effect of BNP-guided care on survival. Brunner-La Rocca *et al*^6^ analysed seven RCTs in patients (n=1580) with HFrEF and four RCTs in patients (n=296) with HFpEF. In younger patients (<75 years), they found the strongest evidence of a beneficial effect of BNP-guided care among patients with HFrEF (HR 0.68 (95% CI 0.48 to 0.96); n=881), while the evidence was weaker for younger patients with HFpEF (HR 0.76 (0.29 to 1.96); n=96). In older patients (≥75 years) with HFrEF, the evidence was inconclusive (HR 0.87 (0.65 to 1.16); n=850), but did not exclude the possibility of a clinically important effect.

Long-term follow-up is unavailable for most RCTs. In the TIME-CHF RCT,^32^ BNP-guided care ceased at 18 months; over a 5-year follow-up period, the study found a non-significant trend for improved survival in younger HFrEF patients with treatment guided by BNP (HR 0.62 (0.37 to 1.03)). Very few patients were followed-up for the full 5 years. In our primary analyses, we assumed that the relative effect of BNP-guided care would end after 4 years. However, it is plausible that BNP-guided care becomes ineffective or less effective before 4 years if, for example, compliance with care decreases or the efficacy of care decreases with age. Equally, the relative effect may extend beyond 4 years. To test the importance of this assumption, we performed sensitivity analyses assuming that the relative effect and cost of BNP-guided care cease at 2 years (SA2) or that the relative effect extends for the lifetime of patients (SA3).

### Baseline probabilities and relative effects: hospitalisation

In the clinically guided group, we used survival analysis of CPRD-ONS linked data, as described earlier, to estimate the monthly hazard rate of all-cause hospitalisation. We then applied this hazard rate throughout the lifetime of patients in the model. Neither Troughton *et al*^7^ nor Brunner-La Rocca *et al*^6^ reported HRs for all-cause hospitalisation stratified by LVEF status. In the absence of evidence, to estimate the relative effect of all-cause hospitalisation in the BNP-guided group, we used a HR of 0.94 (0.84 to 1.07) for patients with any type of HF as reported in an IPD meta-analysis by Troughton *et al*.^7^ We modelled all-cause hospitalisation in our analyses. This is consistent with our focus on all-cause mortality and allows for the possibility that savings through reduced HF readmissions might be partially offset by more admissions for concomitant disorders or side effects of more intensive HF pharmacotherapy. Cohort data comparing patients with HFrEF versus HFpEF suggest that, after adjustment for covariates, there is negligible difference in the risk of all-cause hospitalisation at 1 year.^25^ Therefore, we used the same risk of all-cause hospitalisation for patients with HFrEF and HFpEF.

### Cost parameters

We included the costs of: (1) BNP and renal testing; (2) BNP-related uptitration of pharmacotherapy; (3) unscheduled outpatient appointments; (4) managing patients with HF in the community; and (5) treating patients with HF in hospital.

The cost of a BNP blood test is £15–25.^29^ We used the top end of this range to include the costs of additional renal function tests. We tested the sensitivity of model results to a 50% decrease (SA4) and 50% increase (SA5) in the cost of BNP testing. Cumulative costs depend on the frequency and duration of BNP monitoring. Based on several RCTs,^16–18^ we assumed 3-monthly BNP testing and that costs of BNP testing and uptitration of medications would cease at 18 months.

There is mixed evidence of the effect of BNP-guided care on drug usage. Some trials^17^ ^18^ ^27^ reported increases in doses of some drugs in the BNP-guided arm, whereas others^16^ ^33^ ^34^ do not. In the TIME-CHF RCT economic evaluation,^11^ medication costs were 12% higher (US$747 vs US$668; p=0.04 (2006 values)) in the BNP-guided arm over an 18-month follow-up period. We used this incremental cost (US$79), inflated to 2013/2014 values and converted to £58.32, to estimate the potential increase in medication costs. BNP-guided care might also increase the number of unscheduled outpatient visits due to increased side effects of pharmacotherapy; however, most trials do not report this outcome. The PRIMA trial^27^ found inconclusive evidence of a higher mean number of unscheduled outpatient appointments in the BNP-guided arm than the clinically guided arm at 2 years (1.4 vs 1.1; p=0.06). We used this estimate and a unit cost of £123 per outpatient appointment.^28^

We used CPRD-HES linked data,^3^ to estimate the age-subgroup specific mean costs (table 2) of NHS care during a 3-month period for patients hospitalised with HF at some point during that period and patients not hospitalised during that period. In brief, we identified 1555 patients in England who died with HF in 2012/2013. Of these patients, 47.4% were female and 60.2% had HF diagnosis in the last 2 years, and the mean (SD) age at death was 83 (10) years. We estimated the cost of medications, primary and hospital healthcare during each 90-day period in the 5 years before death. These analyses found no strong evidence of additional NHS costs for patients with left ventricular dysfunction (mean incremental cost: £234 (95% CI −£113 to £580)), and therefore, we used the same estimates in HFrEF and HFpEF subgroup analyses. All costs were estimated in 2013/2014 GBP£.

### Utility parameters

We conducted a structured search of MEDLINE and EMBASE to identify studies which reported utility scores, preferably using the EQ-5D, in patients with HF stratified by hospitalisation status. We included keywords and medical subject headings for HF, utilities and QALYs. The ASCEND-HF multinational trial^26^ reports utility scores among more than 6000 patients hospitalised with acute decompensated HF and randomised to nesiritide or placebo. EQ-5D-3L scores were collected at baseline, 24 hours, discharge and 30 days. In the placebo arm, the mean (SD) EQ-5D-3L utility scores increased from 0.55 (0.29) at admission to 0.66 (0.26) at 24 hours and 0.77 (0.23) at discharge. EQ-5D-3L scores did not change substantially postdischarge; 0.74 (0.25). We assumed that the utility score at 24 hours was representative of the average utility score (U_h_) of patients with acute decompensation during hospitalisation and that the utility score at discharge was representative of average utility score (U_nh_) among patients with stable HF not-hospitalised. We assumed that these two utility values were independent of monitoring strategy; therefore, any improvement in quality of life from BNP-guided care in the model is the result of reducing the risk of readmission. Evidence from most RCTs,^16^ ^17^ ^27^ ^34^ which have measured quality of life, indicates no difference between patients with BNP-guided versus clinically guided care. We assumed that utility values did not decline with age or differ by LVEF status.

Using the CPRD-HES linked data on the 1555 patients described above, we estimated that patients with HF hospitalised would have a mean (SE) length of stay of 13.21 (0.39) days within a 3-month cycle. Therefore, the mean QALYs gained during a 3-month (ie, 91.31-day) cycle which included a hospitalisation (QALYs_h_) and non-hospitalisation (QALYs_nh_) are:
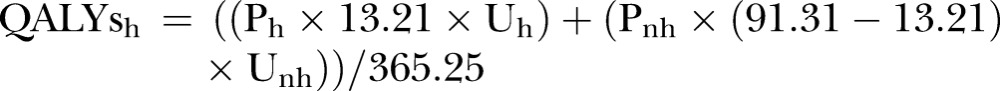

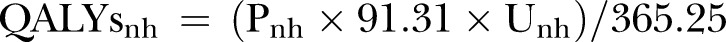


where P_h_ represents the proportion of hospitalised patients at each cycle and P_nh_ represents the proportion of not-hospitalised patients at each cycle.

### Probabilistic sensitivity analysis

We used probabilistic sensitivity analysis (PSA) to estimate 95% CIs around the results.^35^ Monte Carlo simulation was used to draw a randomly selected estimate of each model parameter from the distribution described in tables 1 and 2 and calculate the iNMB. β distributions were used to represent the uncertainty in the probability and utility parameters because these values are typically bounded at zero and one. Log-normal distributions were used to estimate uncertainty in hazard rates and ratios. γ distributions were used to represent the uncertainty in the cost parameters because these values are constrained to be non-negative, but can have skewed distributions. We used 10 000 iterations to empirically estimate the uncertainty surrounding the mean iNMB. We built the model in Microsoft Excel, and programmed in Visual Basic for Applications.

## Results

**Figure 2:**
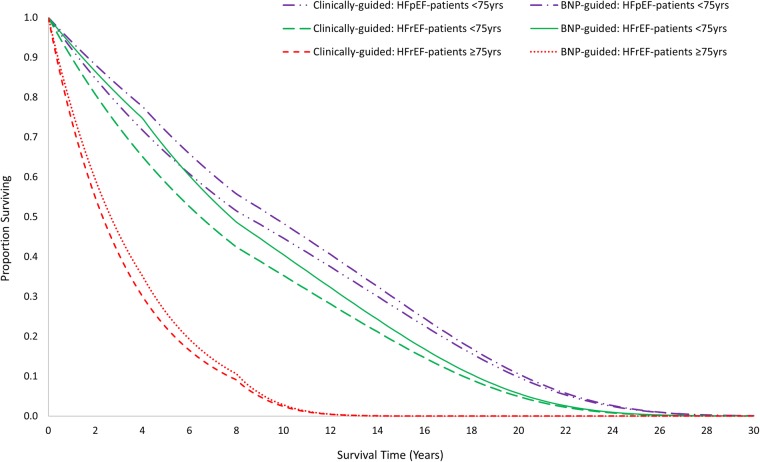
Survival curves for the three patient subgroups.

**Figure 3:**
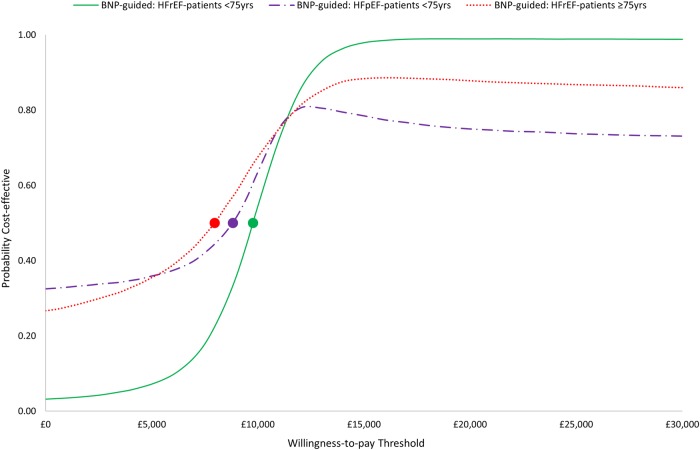
Cost-effectiveness acceptability curves for each of the three patient subgroups in the B-type natriuretic peptide (BNP)-guided care. The circles represent the willingness-to-pay thresholds beyond which the BNP-guided care is most likely to be cost-effective.

Our results indicate that BNP-guided care is more costly, but also more effective, than clinically guided care over the lifetime of younger patients (<75 years) with HFrEF (table 3). The median survival is longer in patients with BNP-guided care (7.75 vs 6.43 years; figure 2). The difference in the mean QALYs is smaller (5.57 vs 5.02; table 3), reflecting the imperfect health of survivors and the discounting of health gained in future years. Lifetime costs are estimated to be higher in patients with BNP-guided care (£63 527 vs £58 139; table 3) as the potential for decreased hospitalisation is more than offset by BNP testing and the costs of healthcare during the extended survival period. The positive iNMB statistic (£5424 (95% CI £987 to £9469); table 3) indicates that BNP-guided care is cost-effective in this subgroup. The CI is broad, primarily due to the uncertainty around the relative effect of BNP-guided care on mortality; however, it does not include zero. There is a high probability (0.99) that BNP-guided care is cost-effective in this subgroup at the willingness-to-pay threshold of £20 000 per QALY (figure 3).

**Table 3:** Cost-effectiveness results in three patient subgroups

	Clinically guided		BNP-guided			
Subgroup	Cost*	QALYs*	Cost*	QALYs*	iNMB† (95% CI)	ICER*
HFrEF patients <75 years	£58 139	5.02	£63 527	5.57	£5424 (£987 to £9469)	£9840
HFpEF patients <75 years	£67 694	5.86	£71 097	6.23	£3155 (−£10 307 to £11 613)	£9066
HFrEF patients ≥75 years	£26 093	2.20	£27 676	2.39	£2267 (−£1524 to £6074)	£8123

*Deterministic analysis.

†Probabilistic sensitivity analysis.

ICER, incremental cost-effectiveness ratio; iNMB, incremental net monetary benefit.

The evidence of cost-effectiveness of BNP-guided care is less strong for the subgroup of younger patients with HFpEF. The median survival (9.54 vs 8.43 years; figure 2) and mean QALYs (6.23 vs 5.86; table 3) are higher in patients with BNP-guided care. However, the iNMB is relatively small with a broad CI spanning zero (£3155 (−£10 307 to £11 613); table 3). Nevertheless, there is a relatively high probability (0.75) that BNP-guided care is cost-effective in this subgroup at the willingness-to-pay threshold of £20 000 per QALY (figure 3).

There is some evidence that BNP-guided care has the potential to be cost-effective among older patients (≥75 years) with HFrEF. However, life expectancy (figure 2) is much shorter in this subgroup than in younger patients; and therefore, the estimated gain in QALYs (2.39 vs 2.20; table 3) and the iNMB are small, and the CI spans zero (£2267 (−£1524 to £6074); table 3). There is a relatively high probability (0.88) that BNP-guided care is cost-effective in this subgroup at the willingness-to-pay threshold of £20 000 per QALY (figure 3).

### Sensitivity analyses

The estimated value of BNP-guided care is sensitive to assumptions about its sustained effect (table 4). If the relative effect and cost of BNP-guided care cease at 2 years (SA2), the incremental costs and QALYs are smaller. However, the conclusion that BNP-guided care is probably cost-effective in younger patients with HFrEF does not change (iNMB: £2834 (£284 to £5079)). If the benefit of BNP-guided care is sustained over patient lifetimes (SA3), the estimated cost-effectiveness (iNMB: £12 275 (£1090 to £24 289)) increases greatly. Substituting a Weibull survival model (SA1) and changing the unit cost of the BNP test (SA4, SA5) had minimal impact on conclusions about cost-effectiveness.

**Table 4:** Sensitivity analyses based on heart failure with reduced ejection fraction patients <75 years

	Clinically guided		BNP-guided			
Sensitivity analysis (SA)	Cost*	QALYs*	Cost*	QALYs*	iNMB† (95% CI)	ICER*
SA1: Weibull form of survival function	£59 025	5.10	£64 939	5.69	£5775 (£936 to £10 073)	£9983
SA2: BNP-guided care cease at 2 years	£58 139	5.02	£61 327	5.33	£2834 (£284 to £5079)	£10 387
SA3: BNP-guided care continue for lifetime	£58 139	5.02	£71 197	6.29	£12 275 (£1090 to £24 289)	£10 274
SA4: Low cost (£12.5) of a BNP test	£58 139	5.02	£63 458	5.57	£5453 (£993 to £9467)	£9714
SA5: High cost (£37.5) of a BNP test	£58 139	5.02	£63 596	5.57	£5303 (£800 to £9328)	£9966

*Deterministic analysis.

†Probabilistic sensitivity analysis.

ICER, incremental cost-effectiveness ratio; iNMB, incremental net monetary benefit.

## Discussion

### Principal findings

We found strong evidence that BNP-guided care is a cost-effective alternative to clinically guided care in younger patients (<75 years) recently hospitalised with HFrEF. This conclusion holds even if the impact of BNP-guided care on mortality is assumed to dissipate after 2 years. The upfront costs of BNP-guided care are justified by improvements in survival. There is no strong evidence that costs of BNP-guided care will be offset by fewer hospitalisations. We also found that BNP-guided care has the potential to be cost-effective in younger patients with HFpEF and older patients (≥75 years) with HFrEF. However, more evidence is required before any firm conclusions can be drawn in these patient subgroups. Relatively, few younger patients with HFpEF are included in RCTs and, therefore, conclusions about cost-effectiveness in this subgroup are tentative. Although a larger number of older patients with HFrEF have participated in RCTs, the effectiveness of BNP-guided care appears to be attenuated in this subgroup and cost-effectiveness remains unproven.

### Strengths and weaknesses

Our analyses used estimates from IPD meta-analyses^6^ ^7^ of ∼2000 patients participating in several RCTs. We investigated the cost-effectiveness of BNP-guided care in subgroups of patients which were not reported in the original RCT publications. Within each subgroup, the meta-analysis preserves the original randomisation; however, comparison of effect sizes between subgroups may be confounded by study as some RCTs contribute no data to some subgroups (eg, HFpEF).^36^ In most RCTs, participants and clinicians were not blind to group allocation, introducing the possibility of performance bias including better ancillary care in the BNP-guided care group unrelated to BNP levels. We conducted the PSA to estimate the uncertainty around our estimates, which allowed us to identify patient subgroups where further evidence is needed. We had insufficient data to estimate the correlation between model parameters which would have allowed a better estimate of the uncertainty in our model results. Deterministic sensitivity analyses allowed us to demonstrate that plausible changes to assumptions about the sustained benefit of BNP-guided care do not alter conclusions that BNP-guided care is cost-effective in younger patients with HFrEF, but are influential in estimating the absolute health benefit for patients.

We used a highly simplified two-state Markov model to track costs and patient outcomes. A model tracking dysfunction (eg, NYHA class) and the probability of hospitalisation and death conditional on dysfunction would provide a more realistic representation of disease progression and the increase in healthcare costs at the end of life. For example, Ieva *et al*^37^ used routine data to model the decrease in time to readmission with each successive admission and the association between age, gender and readmission. The simplicity of our model might lead to poor estimates of cost-effectiveness if BNP-guided care has a large effect on functional decline among survivors. However, several RCTs^16^ ^17^ ^27^ ^34^ provide little evidence of improved quality of life among survivors with BNP-guided care. None of the RCTs provided evidence on utility scores; therefore, we used scores reported elsewhere in the HF literature which may not be representative of patients eligible for BNP monitoring. Our analyses focus on costs to the health service, rather than wider costs falling on social care or patients and families. BNP-guided care will be more cost-effective if, for example, it results in fewer admissions to residential or nursing homes.

### Comparisons with other studies

The TIME-CHF RCT economic evaluation^11^ in HFrEF patients (mean age 76 years) estimated higher costs (US$384 (−$3462 to $4803); after excluding residential costs) and higher QALYs (0.05 (−0.02 to 0.11)) in patients with BNP-guided care at 18 months.^11^ The authors concluded that BNP-guided care had a high probability of being cost-effective, but noted that this probability was lower in older patients. Laramée *et al*^14^ published the only UK-based economic model of BNP-guided care, developing previous work included in the NICE HF clinical guidelines.^8^ Their analysis is based on aggregate, rather than individual patient, data from six RCTs. The structure of their model is similar to ours, but the key model parameter estimates differ. They conclude that BNP-guided care is most probably cost-effective in patients with HFrEF and in younger patients with HF from any cause. An acknowledged limitation of their analysis is that they could not explore cost-effectiveness in patients with HFpEF.

### Implications for clinicians and policymakers

Although BNP tests are relatively cheap, there will be logistical and financial challenges to routine implementation in the UK. If, as in most RCTs, BNP monitoring is conducted in an outpatient setting by physicians skilled in HF care, existing gaps between guidelines and current practice need to be bridged; many patients in the UK do not receive cardiology or HF nurse follow-up.^38^ Cost-effectiveness results based on trials of BNP monitoring by specialists in outpatient settings cannot be simply generalised to primary care. The BATTLESCARRED RCT^16^ compared BNP-guided and clinically guided care in a specialist clinic with usual primary care and found that usual primary care resulted in inferior survival at 1 year. The SIGNAL-HF RCT^34^ which recruited patients with stable HF in primary care found no important improvements in clinical outcomes for patients with BNP-guided care.

Another hurdle to implementing BNP-guided care is that there is little consensus on the optimal frequency, duration and BNP target for monitoring. Developments in medical therapy will also influence the use of monitoring. New drugs, such as Entresto, which inhibits neprilysin and increases the levels of natriuretic peptides, would require different monitoring strategies.^39^ However, NT-proBNP will remain a useful biomarker in this situation.^40^

### Unanswered questions and future research

Most uncertainty in our model is caused by wide CIs surrounding the relative effect of BNP-guided care, particularly in patient subgroups not well represented in RCTs. The ongoing GUIDE-IT trial^41^ will be vital in providing better evidence in older patients with HFrEF and in supporting or refuting existing evidence from smaller RCTs for younger patients with HFrEF. Larger numbers of RCT participants will enable more detailed exploration of other patient subgroups. For instance, comorbidities may explain the lower efficacy of BNP-guided care in older patients.^6^ The potential for harm from aggressive medication titration among elderly patients or those with significant renal dysfunction needs to be explored further before firm conclusions can be drawn in these patient groups.^42^

Despite the high prevalence of HF, there is surprisingly little research on the economic impact on health systems, families and societies.^3^ Future research, particularly on residential care needs, informal care needs and productivity losses due to HF, is needed in order to better judge the economic case for interventions like BNP-guided care.
